# Aquaporin-1 and aquaporin-9 gene variations in sudden infant death syndrome

**DOI:** 10.1007/s00414-020-02493-9

**Published:** 2021-01-18

**Authors:** Siri Hauge Opdal, Linda Ferrante, Torleiv Ole Rognum, Arne Stray-Pedersen

**Affiliations:** 1 Department of Forensic Sciences, Section of Forensic Pathology and Forensic Clinical Medicine, Oslo University Hospital, Oslo, Norway; 2 Institute of Clinical Medicine, University of Oslo, Oslo, Norway

**Keywords:** Aquaporin-1, Aquaporin-9, Genetic predisposition, SIDS, Sudden infant death syndrome

## Abstract

Several studies have indicated that a vulnerability in the development and regulation of brain function is involved in sudden infant death syndrome (SIDS). The aim of this study was to investigate the genes encoding the brain aquaporins (AQPs) AQP1 and AQP9 in SIDS. The hypothesis was that specific variants of these genes are part of the genetic vulnerability predisposing infants to sudden unexpected death. The study included 168 SIDS cases with a median age of 15.5 (range 2–52) weeks and 372 adolescent/adult deceased controls with a median age of 44 (range 11–91) years. In the AQP1 gene, the rs17159702 CC/CT genotypes were found to be associated with SIDS (*p* = 0.02). In the AQP9 gene, the combination of a TT genotype of rs8042354, rs2292711 and rs13329178 was more frequent in SIDS cases than in controls (*p* = 0.03). In the SIDS group, an association was found between genetic variations in the AQP1 gene and maternal smoking and between the 3xTT combination in the AQP9 gene and being found lifeless in a prone position. In conclusion, this study adds further evidence to the involvement of brain aquaporins in SIDS, suggesting that specific variants of AQP genes constitute a genetic predisposition, making the infant vulnerable to sudden death together with external risk factors and probably other genetic factors.

**Supplementary Information:**

The online version contains supplementary material available at 10.1007/s00414-020-02493-9.

## Introduction

Sudden infant death syndrome (SIDS) is defined as the sudden unexpected death of an infant less than 1 year of age, with the onset of the fatal episode apparently occurring during sleep, and which remains unexplained after a thorough investigation, including a complete autopsy and a review of the circumstances of the death and the clinical history [1].

Several studies have indicated that a vulnerability in the development and regulation of brain function is involved in SIDS [2–5]. The most robust neurochemical abnormality involves the medullary serotonergic (5-HT) system, and it has been proposed that an important subset of SIDS infants has serotonergic abnormalities, leading to a failure of protective brainstem responses [6, 7].

Aquaporins (AQPs) are a group of proteins that function as water channels; they facilitate the rapid transport of water and other solutes across the plasma membrane. The three best characterised aquaporins in the brain are AQP1, AQP4 and AQP9 [8]. AQP4 is the main water channel in the brain and spinal cord and it plays a role in brain water homeostasis and neural signal transduction [9, 10]. In mice, it has been reported that a lack of AQP4 expression is paralleled by abnormally high levels of 5-HT in various areas of the brain [11]. This may indicate that a well-functioning water balance is important for the regulation of neurotransmitters, suggesting a possible explanation for the 5-HT imbalances observed in SIDS [7].

In a previous study of the AQP4 gene in Norwegian SIDS, an association was found between rs2075575 CT/TT and SIDS [12], even though this was not replicated in a study of Swiss SIDS cases [13]. An association between a CC genotype (4xCC) in four SNPs (rs17375748, rs1130183, rs12133079 and rs1186688) in the gene encoding the potassium channel Kir4.1 and SIDS has also been reported [14]. This channel is co-expressed with AQP4, and together, they compose a multifactorial unit responsible for maintaining ion and water homeostasis in the brain. The findings in SIDS so far, both concerning the serotonergic network and aquaporins, as well as the lack of clarity with regard to AQP4 and SIDS, may point to related proteins, such as AQP1 and AQP9. AQP1, AQP4 and AQP9 have been shown to have altered levels of expression in several brain disorders in rodents and humans [15].

AQP1 is located in the apical membrane of the choroid plexus, where it is involved in the formation of cerebrospinal fluid but it is also expressed in the capillary endothelium in many organs and in the tubular epithelium of the kidneys [16]. AQP1 has been linked to hydrocephalus conditions and probably plays a role in the development of cytotoxic oedema [10]. Hypoxia and ischaemia have been shown to stimulate increased AQP1 expression both in vitro and in vivo [17]. AQP1 is reported to be upregulated in pathological states such as brain tumours and Alzheimer’s disease [18, 19].

AQP9 is the only aquaglyceroprotein in the brain; this AQP transports glycerol, amino acids and lactate in addition to water. In the brain, AQP9 is expressed in neurons in the brainstem and in the midbrain, and AQP9 is enriched in the inner mitochondrial membrane [20]. Its level of expression is under the control of insulin, and it is believed that AQP9 is involved in energy metabolism in the brain [21].

The purpose of the present study was to investigate genetic variations in the genes encoding AQP1 and AQP9 in SIDS cases and controls. Our hypothesis is that specific variants of these genes predispose infants to sudden unexpected death.

## Materials and methods

### Individuals

The individuals included in this study consisted of 168 SIDS cases and 372 adolescent/adult controls (Table 1). All cases were collected between 1988 and 2013, and all underwent autopsy at the Department of Forensic Medicine at Oslo University Hospital. The investigation protocol included an evaluation of the circumstances of death, a review of their medical and family history, a total skeletal radiographic examination and a thorough autopsy with extensive histologic and microbiologic examinations, including a neuropathological examination and toxicological analysis. Adolescent/adult controls were consecutively collected in 2001–2011 from medico-legal autopsies of cases originating from the same geographical area as the SIDS cases. All SIDS cases and controls included in this study were of Caucasian ethnicity.

**Table 1:** Survey of the individuals included in this study

	SIDS cases	Controls
Number of cases	168	372
Cause of death	SIDS	169 violent death 133 disease 70 intoxication
Sex (male/female)	108/64	265/107
Age (median, range)	15.5 weeks (2–52 weeks)	44 years (11–91 years)
Nicotine exposure	82/27/59^a^	0/0/372^a^
Found dead prone	89/58/21	0/0/372
Infection prior to death	68/77/23	0/0/372

^a^Number of cases: yes/no/unknown

The SIDS cases were classified according to the San Diego definition [1], applying the criteria used in the Nordic SIDS study [22, 23]. After the genetic analyses, three cases with medium-chain acyl-CoA dehydrogenase A985G mutations and six cases with mutations in long QT syndrome-related genes were excluded. A total of 135 cases were classified as pure SIDS, corresponding to category I SIDS according to the San Diego definition. Another 33 cases were classified as category II SIDS in the San Diego definition, corresponding to undetermined in the new classification recommendations [24]. In these cases, the post-mortem investigation revealed findings of slight infection (*n* = 19), insignificant findings in the brain (*n* = 8) or findings in skeletal muscle that might indicate metabolic disease (*n* = 6); however, these findings were not sufficient to fully explain the cause of death.

Information regarding the risk factors nicotine exposure, position of the infant when found lifeless and any known infection prior to death was obtained from the autopsy reports or directly from the parents using a questionnaire (Table 1). The parents received the questionnaire via mail one to several months after the infant’s death, and participation was voluntary [25]. Therefore, some information is missing for some of the cases.

### Gene analyses

**Fig 1:**
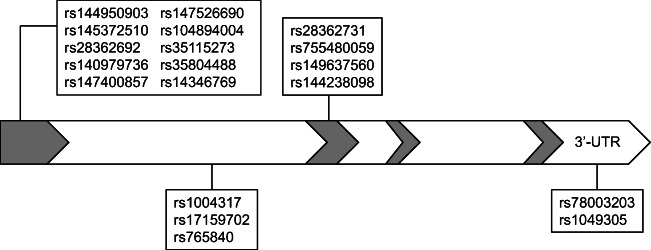
Schematic view of the AQP1 gene with the location of the investigated SNPs indicated. The grey colour identifies exon 1-4

**Fig 2:**
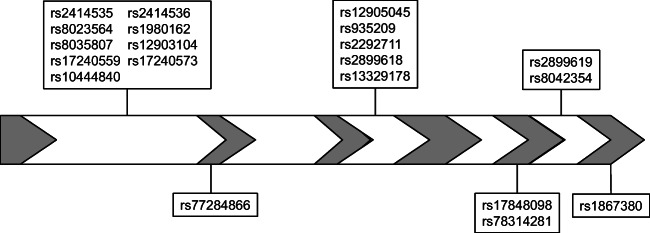
Schematic view of the AQP9 gene with the location of the investigated SNPs indicated. The grey colour identifies exon 1-6

A total of 39 single nucleotide polymorphisms (SNPs) were selected for analysis in the genes encoding AQP1 (located at chromosome 7, 19 SNPs) and AQP9 (located at chromosome 15, 20 SNPs) (Figs. 1 and 2; online resource 1, 2). There were no interactions between the genes or any conjoined genes. The selection of SNPs was based on these criteria: tag SNPs, SNPs recognised to influence the function or production of the gene, and SNPs resulting in amino acid shifts. The online databases used were The International Genome Sample Resource (www.internationalgenome.org), The University of California Santa Cruz Genome Browser (genome.ucsc.edu) and the dbSNP database provided by the National Centre for Biotechnology Information, USA (www.ncbi.nlm.nih.gov). In addition, literature searches for relevant studies, including AQP1 and AQP9, were performed using PubMed, and four additional SNPs were included [26–28].

DNA was extracted from blood/spleen using standard methods, including phenol/chloroform extraction, ethanol precipitation and a QIAmp DNA Mini Kit using the BioRobotEZ (Qiagen, Hombrechtikon, Switzerland).

All SNPs were genotyped using the Sequenom MassARRAY system, following the manufacturer’s protocols (Agena Bioscience, Hamburg, Germany). A multiplex assay was designed using Assay designer software version 2.0, which automatically designed the primers. Following polymerase chain reaction (PCR) and shrimp alkali phosphatase treatment, a primer extension reaction was performed to introduce mass differences between the different alleles. The PCR products were dispensed onto a SpectroCHIP with 384 patches. The mass difference was detected using matrix-assisted laser desorption time-of-flight mass spectrometry (MALDI-TOF MS), and the genotypes were determined using the type 3.1 software (Sequenom, Agena Bioscience, Hamburg, Germany). Cluster plots were made according to the signals of the different allele masses using the default parameters set in the software. An independent platform manager performed the scoring in a blinded fashion. The genotyping was performed at CIGENE, University of Life Sciences, Ås, Norway.

### Statistical analyses

The Hardy-Weinberg equilibrium test was performed using a web-based calculator made available through the Online Encyclopedia for Genetic Epidemiology Studies (www.oege.org). The chi-square test was used for the first comparison of gene frequencies between SIDS cases and controls and for correlations between risk factors and the genotype. These analyses were implemented using SPSS version 20.0 (SPSS, Chicago, IL). The genotype distributions were further analysed using the Haplin statistical package, which is an open source R package for genetic association studies available at folk.uib.no/gjessing/genetics/software/haplin [29]. Power analysis was performed using the power calculator available at clincal.com/statistics/.

## Results

### AQP1

In the AQP1 gene, 19 SNPs were investigated (Fig. 1). Of these, 10 SNPs were homozygous for the most common allele in both SIDS cases and controls. Three of the SNPs showed little variation; only one or a few cases were heterozygous and the rest were homozygous for the most common allele. One SNP (rs28362692) was not in Hardy-Weinberg equilibrium in the controls and was therefore excluded. Five SNPs were subjected to further calculations, including rs1004317, rs17159702, rs765840, rs28362731 and rs1049305. The allele frequencies for each SNP are given in Online Resource 1.

When investigating each SNP separately, a difference in genotype distribution between the SIDS cases and controls was found for one SNP, rs17159702 (*p* = 0.036) (Table 3). For this SNP, the C allele and the CC/CT genotypes were more common in SIDS cases than in the controls; 52.1 % of the SIDS cases had these genotypes, compared to 41.5 % of the controls (*p* = 0.02). The relative risk associated with this SNP was calculated to be 1.4 (1.1–1.9) for the C-allele (*p* = 0.01) and 2.1 (1.8–3.7) for the CC genotype (*p* = 0.01). The difference was confirmed when correcting for multiple testing using the Suest commando in Haplin (*p* = 0.045 for alleles). This test computes a joint overall *p* value based on aggregating the individual *p* values and then correcting for multiple testing, and it was performed on the five SNPs selected for further studies. In addition, there was a tendency for the GG/AG genotypes of rs1004317 to be more frequent in SIDS cases than in controls (*p* = 0.047) (Table 2).

**Table 2:** AQP1 allele variants associated with SIDS

SNP	Genotype	SIDS	Controls	*p* value
rs1004317	GG/AG AA	114 (67.9 %)^*^ 54 (32.1 %)	216 (58.9 %) 151 (41.1 %)	0.047^**^
rs17159702	CC CT TT	17 (10.2 %) 70 (41.9 %) 80 (47.9 %)	23 (6.2 %) 131 (35.3 %) 217 (58.5 %)	0.036^***^

^*^Percentage of the total cases

^**^Chi-square test, 2 × 2 table

^***^Chi-square test, 3 × 2 table

When adding known risk factors, we found that the TT genotype of rs17159702 and the AA/AG genotypes of rs1004317 were more frequent in SIDS cases exposed to maternal smoking than in SIDS cases not exposed (*p* = 0.014 and *p* = 0.004, respectively) (Table 3). No other associations between SNPs in the AQP1 gene and the risk factors for SIDS were found.

**Table 3:** AQP1 genotype frequency in SIDS according to exposure to maternal smoking

SNP	Genotype	Exposed	Not exposed	*p* value^**^
rs1004317	AA/AG GG	72 (87.8 %)^*^ 10 (12.2 %)	17 (63 %) 10 (37 %)	0.004
rs17159702	CC/CT TT	38 (46.9 %) 43 (53.1 %)	20 (74.1 %) 7 (25.9 %)	0.014

^*^Percentage of the total cases

^**^Chi-square 2 × 2 table

### AQP9

In the AQP9 gene, 20 SNPs were investigated (Fig. 2). Amongst these SNPs, one SNP (rs17848098) was homozygous for the most common allele in both the SIDS cases and controls, whilst two SNPs, rs77284866 and rs78314281, showed little variation in the investigated populations. These SNPs were homozygous for the most common allele, with the exception of a few cases found to be heterozygous. All SNPs were in Hardy-Weinberg equilibrium in the controls, and 17 polymorphic SNPs were subjected to further calculations. The allele frequencies for each SNP are given in Online Resource 2.

When investigating each SNP separately, the TT genotypes of rs8042354, rs2292711 and rs13329178 tended to occur more frequently in SIDS cases than in controls (Table 4). Combined, 56 % of the SIDS cases had a 3xTT genotype combination, compared to 46 % of the controls (*p* = 0.03).

**Table 4:** AQP9 allele variants found to be associated with SIDS

SNP	Genotype	SIDS	Controls	*p* value^**^
rs2292711	TT TC/CC	99 (59.3 %)^*^ 68 (40.7 %)	189 (51.4 %) 179 (48.6 %)	0.09
rs13329178	TT TA/AA	101 (60.1 %) 67 (39.9 %)	190 (51.4 %) 180 (48.6 %)	0.06
rs8042354	TT TA/AA	101 (60.1 %) 67 (39.9 %)	188 (50.8 %) 182 (49.2 %)	0.045
Combined	3 x TT other	94 (56.0 %) 74 (46.0 %)	171 (46.0 %) 201 (54.0 %)	0.032

^*^Percentage of the total cases

^**^Chi-square 2 × 2 table

Considering known risk factors for SIDS, 56 of the 82 SIDS cases found lifeless in a prone position (71 %) had the 3xTT genotype combination, compared to 23 of the 52 SIDS cases found lifeless supine or on their side (44 %) (*p* = 0.006). No other associations between SNPs in the AQP9 gene and risk factors for SIDS were uncovered.

### Power analysis

The number of SIDS cases and controls included in this study has the ability to detect a difference in the minor allele frequency of 15 % with a power between 70 and 80 % at the 5 % level of significance.

## Discussion

In this study, it was found that specific variants of the gene encoding AQP1 were more frequent in SIDS cases than in controls (Table 2). AQP1 is present at an early stage in the developing human foetus, and during the early foetal period (8–12 weeks), the third and fourth ventricles of the choroid plexus show strong apical AQP1 staining [30]. This indicates that AQP1 functions as a water channel starting in early foetal life and is thus of functional importance in the developing brain. We found that specific genotypes in two SNPs in the gene encoding AQP1 tended to be more frequent in SIDS cases than in controls (Table 3). Taking into account the essential role of a properly developing choroid plexus and cerebrospinal fluid in the foetal brain, one might speculate that AQP1 gene variations are contributing to a vulnerability to sudden death.

Maternal smoking is a well-known risk factor for SIDS [31]. Nicotine affects the brain through nicotinic acetylcholine receptors (nAChRs), and it has been shown that these receptors are present in the human choroid plexus [32]. Thus, nicotine has the ability to exert site-specific effects on choroid plexus mechanisms and thereby modulate the function of this tissue [32]. A high incidence of histologic/immunohistochemical alterations in the fourth ventricle of the choroid plexus in SIDS has been reported, and these alterations are significantly related to maternal smoking [33]. APQ1 is located at the apical membrane of the choroid plexus, and it is interesting that the present study revealed an association between genetic variations in the AQP1 gene and maternal smoking in SIDS (Table 4). Our observations strengthen the theory by Lavezzi et al. that some SIDS cases have a vulnerability in the choroid plexus that could be associated with distortions in the cerebrospinal fluid volume, its content and its flow dynamics [33].

The present study found an association between two SNPs in the AQP1 gene and exposure to maternal smoking (Table 3). It is, however, intriguing that although the CT/TT genotypes of rs17159702 were more common in SIDS cases than in controls, the only genotype found to be associated with maternal smoking was the TT genotype. However, for SIDS to occur, it seems that multiple genetic and environmental risk factors must be present. One might therefore speculate that, as maternal smoking is a strong environmental risk factor for SIDS, the death of infants exposed to nicotine depends to a lesser extent on their genetic predisposition. Another explanation may be that the discovered variant acts as a phenotype modifier rather than being a strong genetic predisposing factor for SIDS.

The present study also found specific variants in the AQP9 gene that were more frequent in SIDS cases than in controls (Table 4). The expression of AQP9 has been described in several brain structures, including catecholaminergic neurons [34]. These neurons are known to be involved in energy balance, and the presence of AQP9 may facilitate the diffusion of glycerol that serves as an energy substrate. AQP9 is also present in astrocytes, and it has been shown that a reduction in AQP9 expression induces a decrease in glycerol uptake and changes in astrocyte energy metabolism [35]. In this study, it was found that the combination of a TT genotype in three SNPs in the AQP9 gene was more frequent in SIDS cases than in controls (Table 4). In addition, the 3xTT genotype combination was more frequent in SIDS cases found lifeless in a prone position compared to cases found lifeless supine. One might speculate that part of the risk for SIDS induced by prone sleeping is linked to energy metabolism and a low energy supply due to a lower feeding frequency during the nighttime than during the daytime.

In addition to prone sleeping and maternal smoking, a minor infection in the week prior to death is a risk factor for SIDS [25]. Amongst the cases included in this study, approximately half of the cases had a slight upper airway infection during the last week prior to death. This is more frequent than expected in a series of 100 infants seen in our department for paternity testing, where 26 % either had signs of or had a history of a recent common cold [25]. However, we did not find any association between the investigated SNPs and signs of infection in the SIDS cases. This may indicate that a susceptibility to infection is more likely due to variations in genes encoding components of the immune response than in genes encoding brain aquaporins.

It is interesting that several SNPs in aquaporin genes are associated with known external risk factors for SIDS. Previously, rs2075575 CT/TT in the AQP4 gene was reported to be associated with maternal smoking, whilst a combination of 4xCC in rs17375748, rs1130183, rs12133079 and rs1186688 in the gene encoding Kir4.1 was reported to be associated with the prone sleeping position [12, 14]. The present study found that allelic variants of two SNPs in the AQP1 gene are associated with maternal smoking and that three SNPs in the AQP9 gene are associated with a prone sleeping position (Tables 3 and 4). Taken together, these findings fit with the theory of a fatal triangle in SIDS [36]. This theory states that infants may die from SIDS when three conditions occur at the same time: a genetic predisposition, a vulnerable developmental stage in the central nervous system and/or immune system and a triggering event such as maternal smoking or prone sleeping. Given that all three occur, the mechanism of death is a vicious circle starting with trigger events and ending with brain oedema, hypoxia, coma and death [37].

There are a vast number of papers investigating a possible genetic predisposition to SIDS. To date, genes involved in cardiac function, the regulation of the immune system, and brain development and regulation have emerged as the most important [38–40]. A recent study, performing next-generation sequencing of cardiac arrhythmia genes, reported likely pathogenic variants in 15 % of the investigated SIDS cases [40]. With regard to the immune system, the most important genes seem to be those encoding interleukins, in particular IL-6, IL-10 and TNFα, whilst in the serotonergic network, the most promising gene seems to be the gene encoding the serotonin transporter 5-HTT [3, 38]. It is interesting that TNFα-mediated signalling has been reported to upregulate AQP4 and that AQP4 participates in the regulation of serotonergic neurotransmission in different brain regions [11, 41].

In conclusion, this study adds further evidence to the involvement of brain aquaporins in SIDS, suggesting that genetic variations in AQP1 and AQP9 together with the AQP4 complex constitute a genetic predisposition making the infant vulnerable to sudden death in concert with external risk factors and probably other genetic factors.

## Supplementary Information

ESM 1 (PDF 97 kb)

ESM 2 (PDF 113 kb)

## Data Availability

The datasets generated and analysed during the current study are available from the corresponding author on reasonable request.
