# Supplementary material for: Aquaporin-1 and aquaporin-9 gene variations in sudden infant death syndrome
Source: Int J Legal Med. 2021 Jan 18;135(3):719–25. doi: 10.1007/s00414-020-02493-9 (PMC8036210; doi:10.1007/s00414-020-02493-9)
Supplement: Supplementary file 1 — (PDF 97 kb) [file 414_2020_2493_MOESM1_ESM.pdf]

Aquaporin-1 and aqpuporin-9 gene variation in sudden infant death syndrome

International Journal of Legal Medicine

Siri Hauge Opdal, Linda Ferrante, Torleiv Ole Rognum, Arne Stray-Pedersen

Corresponding author: Siri Hauge Opdal, Department of Forensic Sciences, Oslo University Hospital, Oslo, Norway, siropd@ous-hf.no

Online Resource 1

Single nucleotide polymorphism (SNP) frequencies in the AQP1 gene, located on chromosome 7, in SIDS cases and controls. The chromosome position is given according to GRCh38

SNP	Chromosome position	Localization	Amino acid change, reason for inclusion	Genotyping result			p-value Chi-square[c]
				Genotype	SIDS N[a] (%)[b]	Controls N (%)	
rs144950903	30911913	exon 1	ala2-thr	GG	114 (100)	328 (100)	
rs147526690	30911997	exon 1	gly30-ser	GG	168 (100)	372 (100)	
rs145372510	30912018	exon 1	tyr-37his	TT	168 (100)	370 (100)	
rs104894004	30912022	exon 1	pro38-leu	CC	168 (100)	372 (100)	
rs28362692	30912043	exon 1	ala45-val	CC<br>CT<br>TT	153 (91.1)<br>14 (8.3)<br>1 (0.6)	337 (91.8)<br>26 (7.1)<br>4 (1.1)	0.76
rs35115273	30912063	exon 1	val52-leu	GG	167 (100)	372 (100)	
rs140979736	30912126	exon 1	ala73-thr	GG	168 (100)	372 (100)	
rs35804488	30912228	exon 1	val107-ile	GG<br>AG	168 (100)<br>0	371 (99.7)<br>1 (0.3)	
rs147400857	30912229	exon 1	val107-gly	TT<br>GT	168 (100)	369 (99.2)<br>3 (0.8)	
rs143467679	30912252	exon 1	ile115-val	AA	168 (100)	372 (100)	

rs1004317	30917243	intron 1	Tag SNP	AA<br>AG<br>GG	54 (32.1)<br>81 (48.2)<br>33 (19.6)	151 (41.1)<br>160 (43.6)<br>56 (15.3)	0.12
rs17159702	30919387	intron 1	Tag SNP	TT<br>CT<br>CC	80 (47.9)<br>70 (41.9)<br>17 (10.2)	217 (58.5)<br>131 (35.3)<br>23 (6.2)	0.046
rs765840	30919869	intron 1	Tag SNP	TT<br>AT<br>AA	133 (79.2)<br>34 (20.2)<br>1 (0.6)	317 (85.9)<br>50 (13.6)<br>2 (0.5)	0.14
rs28362731	30922175	exon 2	gly165-asp	GG<br>AG<br>AA	157 (93.5)<br>11 (6.5)	347 (93.5)<br>23 (6.5)<br>1 (0.3)	0.79
rs755480059	30922590	exon 2	asn192-lys	CC	165 (100)	370 (100)	
rs149637560	30923561	exon 2	gly248-ser	GG<br>GA	167 (99.4)<br>1 (0.6)	368 (100)<br>2 (0.7)	
rs144238098	30923577	exon 2	tyr253-cys	AA	167 (100)	370 (100)	
rs78003203	30923911	3'-UTR[d]	Tag SNP	CC	166 (100)	370 (100)	
rs1049305	30924207	3'-UTR	Affect expression	GG<br>GC<br>CC	55 (32.9)<br>83 (49.7)<br>29 (17.4)	144 (39.6)<br>167 (45.9)<br>53 (14.6)	0.32

[a]: Number of cases

[b]: Percent of number of cases with successful genotyping

[c]: 3x2 table

[d]: Untranslated region
