# Supplementary material for: Aquaporin-1 and aquaporin-9 gene variations in sudden infant death syndrome
Source: Int J Legal Med. 2021 Jan 18;135(3):719–25. doi: 10.1007/s00414-020-02493-9 (PMC8036210; doi:10.1007/s00414-020-02493-9)
Supplement: Supplementary file 2 — (PDF 113 kb) [file 414_2020_2493_MOESM2_ESM.pdf]

Aquaporin-1 and aqpuporin-9 gene variation in sudden infant death syndrome
International Journal of Legal Medicine
Siri Hauge Opdal, Linda Ferrante, Torleiv Ole Rognum, Arne Stray-Pedersen
Corresponding author: Siri Hauge Opdal, Department of Forensic Sciences, Oslo University Hospital, Oslo, Norway, siropd@ous-hf.no

Online Resource 2

Single nucleotide polymorphism (SNP) frequencies in the AQP9 gene, located on chromosome 15, in SIDS cases and controls. The chromosome position is given according to GRCh38.

SNP	Chromosome position	Localization	Amino acid change, reason for inclusion	Genotyping result			p-value Chi-square[c]
				Genotype	SIDS N[a] (%)[b]	Controls N (%)	
rs2414535	58141091	intron 1	Tag SNP	GG	59 (35.1)	112 (30.5)	0.57
				GC	79 (47)	186 (50.7)	
				CC	30 (17.9)	69 (18.8)	
rs2414536	58141170	intron 1	Tag SSP	CC	61 (37.2)	116 (31.6)	0.42
				GC	75 (45.7)	188 (51.2)	
				GG	28 (17.1)	63 (17.2)	
rs8023564	58145626	intron 1	Tag SNP	CC	60 (38.7)	125 (36.5)	0.48
				CT	66 (42.6)	164 (48)	
				TT	29 (18.7)	53 (15.5)	
rs1980162	58146336	intron 1	Tag SNP	CC	49 (29.3)	87 (23.4)	0.31
				GC	77 (46.1)	192 (51.6)	
				GG	41 (24.6)	93 (25)	

rs8035807	58147995	intron 1	Tag SNP	TT<br>CT<br>CC	137 (83)<br>27 (16.4)<br>1 (0.6)	287 (81.1)<br>64 (19.1)<br>3 (0.8)	0.85
rs12903104	58151567	intron 1	Tag SNP	TT<br>CT<br>CC	89 (53)<br>64 (38.1)<br>15 (8.9)	221 (59.4)<br>131 (35.2)<br>20 (5.4)	0.19
rs17240559	58155913	intron 1	Tag SNP	GG<br>GA<br>AA	104 (61.9)<br>55 (32.7)<br>9 (5.4)	219 (59.8)<br>124 (33.9)<br>23 (6.3)	0.86
rs17240573	58156112	intron 1	Tag SNP	GG<br>AG<br>AA	62 (36.9)<br>72 (42.9)<br>34 (20.2)	113 (30.6)<br>176 (47.7)<br>80 (21.7)	0.35
rs10444840	58158075	intron 1	Tag SNP	GG<br>AG<br>AA	65 (38.7)<br>72 (42.9)<br>31 (18.5)	127 (34.5)<br>173 (47)<br>68 (18.5)	0.61
rs77284866	58166690	exon 2	cys43-trp	TT	168 (100)	372 (100)	
rs12905045	58169615	intron 3	Tag SNP	CC<br>CA<br>AA	108 (64.3)<br>55 (32.7)<br>5 (3)	233 (63.5)<br>116 (31.6)<br>18 (4.9)	0.59
rs935209	58172121	intron 3	Tag SNP	TT<br>CT<br>CC	89 (53)<br>67 (39.9)<br>12 (7.1)	210 (57.1)<br>137 (37.2)<br>21 (5.7)	0.62
rs2292711	58172748	intron 3	Tag SNP	TT<br>CT<br>CC	99 (59.3)<br>57 (34.1)<br>11 (6.6)	189 (51.4)<br>149 (40.5)<br>30 (8.2)	0.23
rs2899618	58175793	intron 3	Tag SNP	AA<br>AG	43 (25.7)<br>93 (55.7)	100 (27)<br>178 (48.1)	0.18

				GG	31 (18.6)	92 (24.9)	
rs13329178	58176542	intron 3	Tag SNP	TT<br>AT<br>AA	101 (60.1)<br>55 (32.7)<br>12 (7.1)	190 (51.4)<br>155 (41.9)<br>25 (6.8)	0.13
rs17848098	58179192	exon 5	ala187-val	GG	172 (100)	370 (100)	
rs78314281	58179271	exon 5	cys213-trp	TT	172 (100)	369 (100)	
rs2899619	58180007	intron 5	Tag SNP	CC<br>CT<br>TT	39 (23.4)<br>91 (54.5)<br>37 (22.2)	114 (30.8)<br>176 (47.6)<br>80 (21.6)	0.14
rs8042354	58180864	intron 5	Tag SNP	TT<br>AT<br>AA	101 (60.1)<br>55 (32.7)<br>12 (7.1)	188 (50.8)<br>157 (42.4)<br>25 (6.8)	0.10
rs1867380	58184082	exon 6	thr279-ala	GG<br>AG	154 (92.8)<br>12 (7.2)	314 (92.4)<br>26 (7.6)	0.87
